# Can Prenatal and Postnatal Cell Phone Exposure Increase Adverse Maternal, Infant and Child Outcomes?

**DOI:** 10.1055/s-0041-1736173

**Published:** 2021-12-06

**Authors:** Farzaneh Ashrafinia, Somayeh Moeindarbari, Parisa Razmjouei, Masumeh Ghazanfarpour, Mona Najaf Najafi, Amir Ali Moodi Ghalibaf, Fatemeh Abdi

**Affiliations:** 1 Department of Midwifery, School of Nursing and Midwifery, Kerman University of Medical Sciences, Kerman, Iran; 2 Student Research Committee, Kerman University of Medical Sciences, Kerman, Iran; 3 Department of Gynecology and Obstetrics, Shahid Faghihi Hospital, Shiraz University of Medical Sciences, Shiraz, Iran; 4 Clinical Research Unit, Mashhad University of Medical Sciences, Mashhad, Iran; 5 Student Research Committee, Faculty of Medicine, Mashhad University of Medical Sciences, Mashhad, Iran; 6 Non-communicable Diseases Research Center, Alborz University of Medical Sciences, Karaj, Iran

**Keywords:** cell phone, maternal outcome, infant outcomes, celular, resultado materno, resultados em crianças

## Abstract

**Objective** To determine the association between maternal mobile phone use and adverse outcomes in infants, children, and mothers.

**Method** In March 202, we conducted a search on the MEDLINE, Embase, and Scopus databases. Data extraction and an assessment of the quality of the studies were performed by two authors. The quality of the studies was assessed using the checklist of the Newcastle-Ottawa scale.

**Results** Studies assessing behavioral problems in infants aged 6 to 18 months reported null findings. However, an increased risk of emotional and behavioral disorders was observed in children aged between 7 and 11 years whose mothers had been exposed to cell phones. The findings regarding the association between maternal cell phone exposure and adverse outcomes in children aged 3 to 5 are controversial. A study found a significant association between the call time ( *p* = 0.002) or the history of mobile phone use (in months) and speech disorders in the children ( *p* = 0.003). However, another study found that maternal cell phone use during pregnancy was not significantly associated with child psychomotor and mental developments. Inconclusive results were observed about the adverse outcomes in fetuses, such as fetal growth restriction or t scores for birth weight in cell phone users as opposed to non-users. On the contrary, the children of mothers who were cell phone users had a lower risk of scoring low on motor skills. Similar results were observed regarding the adverse outcomes of cell phone use in infants, such as fetal growth restriction or low birth weight, and the risk of preeclampsia was lower among subjects with medium and high cell phone exposure, as opposed to those with low exposure.

**Conclusion** Studies on behavioral problems have reported different postnatal results, such as null findings among infants and a positive association in children.

## Resumo

**Objetivo** Determinar a associação entre o uso de telefone celular pela mãe e os resultados adversos em recém-nascidos crianças e mães.

**Método** Em março de 2020 realizou-se uma pesquisa nas bases de dados MEDLINE, Embase e Scopus. A extração de dados e avaliação da qualidade dos estudos foram realizadas por dois autores. A qualidade dos estudos foi avaliada por meio da lista de verificação da escala Newcastle-Ottawa.

**Resultados** Estudos que avaliavam problemas comportamentais em recém-nascidos de 6 a 18 meses relataram resultados nulos. No entanto um risco aumentado de transtornos emocionais e comportamentais foi observado em crianças de 7 a 11 anos de idade cujas mães foram expostas a telefones celulares. Os resultados relacionados à associação entre a exposição materna a celulares e resultados adversos em crianças de 3 a 5 anos são controversos. Um estudo encontrou associação significativa entre o tempo de ligação ( *p* = 0.002) ou o histórico de uso de celular (em meses) e distúrbios de fala nas crianças ( *p* = 0.003). No entanto outro estudo descobriu que o uso de telefone celular pela mãe durante a gravidez não estava significativamente associado ao desenvolvimento psicomotor e mental da criança. Resultados inconclusivos foram observados com relação aos resultados adversos de fetos como restrição de crescimento intrauterino ou valores de t para peso ao nascer em usuárias de telefone celular em oposição a não usuárias. Pelo contrário os filhos de mães usuárias de telefone celular apresentaram menor risco de pontuação baixa em habilidades motoras. Resultados semelhantes foram observados com relação a resultados adversos em recém-nascidos como restrição de crescimento intrauterino ou valores de peso ao nascere o risco de pré-eclâmpsia foi menor em indivíduos com exposição média e alta a celulares em oposição àqueles com baixa exposição.

**Conclusão** Estudos sobre problemas comportamentais relataram resultados diferentes no pós-natal como achados nulos em recém-nascidos e associação positiva em crianças.

## Introduction

One of the most important devices that has seen a dramatic growth in recent years is the cell phone. 1 Research shows that cell phones could expose a user to radiofrequency electromagnetic fields (RF-EMFs). 2 Excessive mobile phone use in Japan is not limited to students, and can be used in adult women, even during the prenatal period. 3 Cell phone based interventions and monitoring are used in the field of maternal and maternity health care. 4

Research has shown the safety of the short-term exposure to RF-EMFs in adults, while long term exposure have not been conclude. Fetuses and children, as opposed to adults, may be more vulnerable to the effects of the long-term exposure to RF-EMFs on human health. 5 Studies have questioned the theory of the thermal effect induced by cell phones because the rate of absorption of cell phone RF by the pregnant uterus is not high enough to raise the body temperature. 6 7 8 9 10 11 12 13 There is still ongoing research on the non-thermal effects of RF radiation (RFR).

According to a study 6 conducted in rats, the exposure of mothers to cell phones may be associated with behavioral complications in the offspring, though no side effects have been reported. There are divergent epidemiological findings regarding the prenatal exposure of mothers to cell phones and null results in the earlier stages of an infant's life; however, a positive relationship has been reported at later stages, except for a study that employed a prospective questionnaire. 7 Researchers have explored the health consequences of the exposure to cell phone RF, but there is a need for further studies to draw definitive conclusions. 5 Accordingly, a systematic review is needed to summarize and scrutinize all the findings in this field to help the clinical practice and reveal the gap in the existing evidence.

## Methods

### Literature Search and Selection Criteria

In March 2020, a literature search was conducted on the MEDLINE, Embase, and Scopus databases using the terms *radiofrequency* , *RF* , *RF-EMFs* , *phone* , *mobile phone* , *cell phone* , *electromagnetic field* , *electromagnetic waves* , *EMF* , *EMW* , *children* , and *behavior* . After reading the title and abstract of all studies during the screening stage, all full-length articles were carefully reviewed by two independent researchers to check the inclusion and exclusion criteria. Any disagreement between the two researchers was settled through consensus. The inclusion criterion was any study on the association of maternal cell phone use and infant and maternal outcomes.

### Data Extraction and Statistical Analysis

The following data was extracted from the studies and recorded in a form designed by the research team: author, year, country, study design, population, study duration, source of information, disorder diagnosis instrument, outcomes (relative risk [confidence interval, CI]), and covariates ( Table 1 ). The necessary adjustments were made, and disagreements were resolved by discussion to reach a decision.

**Table 1:** Summarized characteristics of studies reviewed

Author	Year	Country	Study design	Population	Study population	Study duration	Source of information	Disorder diagnosis instrument	Comparison groups	Outcome: relative risk (confidence interval)	Covariate adjustment
Papadopoulou et al. 4	2017	Norway	Cohort	45,389 for child's language, communication, and motor skills; 17,310 for neurodevelopmental outcomes	Pregnant women at 17–18 weeks of gestational age (used cellphone according to the cellphone use frequency questionnaire) and their children at 3 and 5 years of age	1999–2014	Population-based	Dale and Bishop grammar rating/ ASQ/ CDI	Pregnant women according to cellphone use frequency questionnaire (no use)	Low sentence complexity at 3 years: 0.83 (0.77–0.89); specified language skills: 0.86 (0.79–0.93); low risk of having incomplete grammar: 0.69 (0.59–0.81); modarate language delay: 0.49 (0.30–0.80)	Maternal age (years), maternal education (≤ 12 years/13– 16 years/≥ 17 years), parental income (both parents low income/either parent high income/both parents high income), parity (primiparous/multiparous), maternal occupation (public sector or military/private sectors or sel-femployed/ other), computer screen use during pregnancy (yes/no), marital status (living with partner/other), smoking prior to and during pregnancy (no/occasionally/ daily), alcohol consumption prior to and during pregnancy (never or < 1 time per month/1–3 times per month/ ≥1 time per week), use of folic acid supplements during pregnancy (yes/no), prepregnancy body mass index (< 18.5, 18.5–24.9, 25–29.9, ≥ 30 kg/m ^2^ ), type of delivery (c-section/vaginal), and the length of gestation (in weeks)
Divan et al. 10	2012	USA	Cohort	28,745	Pregnant women and their children at 7 years of age	1996–2002	Danish Medical Birth Registry	Mother's history of psychiatric problems (self-reported from Age-7 Questionnaire) / SDQ	Pregnant women and their children at 7 years of age without exposure during the same period of the study group.	Prenatal and postnatal exposure: 1.5 (1.4–1.7); prenatal exposure: 1.4 (1.2–1.5); postnatal exposure: 1.2 (1.0–1.3)	Child's gender; mother's age at birth; father's age at birth; mother's history of psychiatric problems (self-reported from Age-7 Questionnaire); mother's history of same psychiatric, behavioral, or cognitive problems as child (self-reported from prenatal interviews); father's history of same psychiatric, behavioral, or cognitive problems as child (spousal report from prenatal interviews); socio-occupational status, including High, Mid, Low; prenatal smoking (entire pregnancy, early pregnancy, or not a smoker); prenatal alcohol intake (entire, early, or late pregnancy only, or not at all); and prenatal marijuana use (yes or no); prenatal stress (14-point summary score categorized as low (0–4), medium (5), or high (6–14)); prenatal physical activity (entire, early, or late pregnancy, or no activity); other sources of prenatal ionizing and non-ionizing radiation (such as, X-rays, ultrasound); parity; gestational age; birth weight; postpartum stress (15-point summary score categorized as low (0–3), medium (4), or high (5–15)); child breastfed for at least the first 6 months (yes or no); hours spent with child daily by ages 6 and 18 months; and child in daycare by 18 months
Choi et al. 7	2017	South Korea	Cohort	1,198	Pregnant women and their children up to 3 years of age	2006–2010	Mothers and Children's Environmental Health (MOCEH) study Registry	Cell phone use frequency questionnaire/ Blood test for lead level/ BSID-II/ exposure meter	Pregnant women according to cell phone use frequency questionnaire	The psychomotor development index (PDI) and the mental development index (MDI) at 6, 12, 24, and 36 months of age were not significantly associated with maternal mobile phone use during pregnancy. There was also a risk of having decreasing MDI up to 36 months of age, in relation to an increasing average calling time or frequency during pregnancy ( *p* -trend = 0.05 and 0.007 for time and frequency respectively). There was no significant association between child neurodevelopment and prenatal RFR exposure measured	The year of enrollment, center area, and responses to questions concerning maternal age at pregnancy (< 30, 30–34, and ≥ 35 years), household income (< 2,000, 2,000–3,000, and ≥ 3,000 103 Korean won (KRW) per month), whether or not the mother is employed, level of schooling (≤ 12 or > 12 years), and frequency of headset use (never, sometimes, or often to always), gestational age, gender of the infant, birth order, maternal intelligence quotient (IQ), prenatal second-hand smoking exposure, maternal urinary cotinine
Sudan et al. 8	2016	Denmark	Cohort	51,190	Pregnant women and their children at 6 and 8 months, and 7 and 11 years of age	1996–2014	Danish National Birth Cohort (DNBC) Registry	Telephone interview/ Age-7 DNBC questionnaire/ SDQ	Pregnant women according to cell phone use frequency questionnaire (no exposure)	Exposed both prenatally and used cell phones at age 7 years: 1.58 (1.34–1.86); prenatal exposure only: 1.41 (1.20–1.66); age-7 use only: 1.36 (1.14–1.63)	Child's gender, mother's age, mother's and father's history of same psychiatric, cognitive, or behavioral problems as the child, Socio-occupational status (High, Mid, Low); gestational age at birth, mother's prenatal stress, and breastfeeding
Vrijheid et al. 12	2010	Spain	Cohort	530	Pregnant women and their children at 14 monthes of age	2004–2006	Population-based	Cell phone use frequency questionnaire/ BSID-II/ Cattell's intelligence test	Pregnant women according to cellphone use frequency questionnaire	Only small differences in neurodevelopment scores between the offspring of cell phone users and nonusers. Those of users had higher mental development scores and lower psychomotor development scores	Maternal socio-economic status, maternal education, and maternal IQ, mater nal age, maternal smoking at any time during pregnancy, and smoker in the house
Guxens et al. 14	2013	Netherlands	Cohort	2,618	Pregnant women and their children at 5 years of age	2003–2004	Amsterdam-born Children and their Development (ABCD), population -based	Cell phone use frequency questionnaire/ SDQ	Pregnant women according to cell phone use frequency questionnaire (non-user)	< 1 call/day: 2.12 (0.95–4.47) 1–4 calls/day: 1.58 (0.69–3.60) ≥ 5 calls/day: 2.04 (0.86–4.80)	Maternal age, maternal Level of schooling (based on the years after primary school: high (≥ 10 years), medium (6–9 years) and low (≤ 5 years)), maternal country of birth, maternal parity, maternal prepregnancy weight and height, maternal smoking, maternal second-hand smoking at home, maternal alcohol consumption during pregnancy, maternal pregnancy-related anxiety, and maternal anxiety and depression during pregnancy was obtained by a questionnaire completed by the mother.

Abbreviations: ASQ, Age and Stage questionnaire; BSID-II, Bayley Scales of Infant Development-Revised CDI, Child Development Inventory questionnaire; RFR, radiofrequency radiation; SDQ, Strengths and Difficulties Questionnaire.

d ().

### Quality Assessment of Studies

The quality of the studies was assessed using Newcastle-Ottawa Scale (NOS) checklist, which investigates the selection criteria of cohorts (representativeness of the exposed cohort, selection of the non-exposed cohort, ascertainment of exposure, demonstration that the outcome of interest was not present at the beginning of the study), the comparability, and the outcome ( Table 2 ).

**Table 2:** Quality of studies using the Newcastle-Ottawa Scale checklist

Author	Selection													Comparability		Outcome									
	Represent exposed				Selection non exposed			Ascertainment of exposure				Outcome wasn't at start				Assessment				Follow-up long enough		Adequacy of follow-up			
	Truly represents community	Somewhat represents community	Selected	None	Same community	Different source	None	Secure record	Structured interview	Self-report	None	Yes	No	Most important	Any additional factor	Independent blind	Record linkage	Self-report	None	Yes	No	Complete	Small no bias	Rate of falloff up no description	None
Papadopoulou et al. (2017) 4	*	-	−	−	*	−	−	−	−	*	−	*	−	*	−	−	−	*	−	*	−	*	−	−	−
Divan et al. (2012) 10	*	−	−	−	*	−	−	−	*	−	−	*	−	*	−	−	−	*	−	*	−	*	−	−	−
Choi et al. (2017) 7	*	−	−	−	*	−	−	−	−	*	−	*	−	*	−	−	−	*	−	*	−	*	−	−	−
Sudan et al. (2016) 8	*	−	−	−	*	−	−	−	*	−	−	*	−	*	−	−	*	*	−	*	−	*	−	−	−
Vrijheid et al. (2010) 12	*	−	−	−	*	−	−	−	−	*	−	*	−	*	−	*	−	−	−	*	−	*	−	−	−
Guxens et al. (2013) 14	*	−	−	−	*	−	−	*	−	*	−	*	−	*	−	−	−	*	−	*	−	*	−	−	−

## Results

### Maternal Cell Phone Use and Behavioral Problems in Children

In a study by Sudan et al., 8 mothers of 7-year-old children were asked to complete a questionnaire that investigated prenatal and postnatal cell phone exposure. The children were then followed up until the age of 11, and the authors found an increased risk of developing emotional and behavioral problems by that age.

An odds ratio (OR) of 1.58 (95%CI: 1.34 to 1.86) when children were exposed to both prenatally and used cellphones at age 7 years, OR of 1.41(95%CI: 1.20 to 1.66) for prenatal exposure and an OR of 1.36 (95%CI: 1.14 to 1.63) for the postnatal exposure. 8

Divan et al., 9 in a study with mothers of 13,159 7-year-old children, observed a significant association between behavioral problems in children and prenatal cell phone exposure. The highest OR for behavioral problems was observed in children who had both prenatal and postnatal cell phone exposure (OR = 80; 95%CI: 1.45 to 2.23), followed by prenatal exposure alone (OR = 1.54; 95%CI: 1.32 to 1.81) and postnatal exposure alone (OR = 1.18; 95%CI: 1.01 to 1.38). To account for additional confounders (including variables that show the mother's attention to the health of the child in the early stages of life), Divan et al. 10 conducted a large-scale study on 28,745 mothers of 7-year-old children. The results indicated a connection between behavioral problems and exposure during both periods. The OR was of 1.5 (95%CI: 1.4 to 1.7) for the prenatal and postnatal exposure, of 1.4 (95%CI: 1.2 to 1.5) for the prenatal exposure alone, and of 1.2 (95%CI: 1.0 to 1.3) for the postnatal exposure alone. 10 Zarei et al. 11 conducted a study on mothers of healthy children aged 3 to 5 years, and found a significant association between call time ( *p* = 0.002) or the history of mobile phone use (in months) and speech disorders in children ( *p* = 0.003). However, the strength of the association between cordless phone use ( *p* = 0.528) and speech disorders was weak. 11

Contrary to the aforementioned studies, Papadopoulo et al., 4 in a prospective study on 45,389 mother-child pairs, reported that children whose mothers were cell phone users in the early months of pregnancy had a lower risk of developing low motor skills and 17% had a lower adjusted risk of developing sentence complexity (OR = 0.83; 95%CI: 0.77, 0.89) at the age of 3, as opposed to children whose mothers did not use cell phones, but the difference was not observed in 5-year-old children. An association was also found between maternal cell phone use and the development of low communication skills in children. The risk was 13%, 22% and 29% lower by low, medium and high maternal cell phone use. 4

In another study, 7 maternal cell phone use during pregnancy was found to be significantly associated with the psychomotor development index (PDI) and mental development index (MDI) in infants and children at 6, 12, 24, and 36 months of age. However, in children exposed to high maternal blood lead level (BLL) in utero, an increased risk of low MDI was observed with an increasing number of calls a day. According to Vrijheid et al., 12 the children of cell phone users had higher mental development scores and lower psychomotor development scores compared with those of non-users at 14 months of age. However, the difference was slight. A significant difference was only observed between the children of compulsive users and those of non-users. The highest decrease in psychomotor scores (5.6 points [95% confidence interval 10.7 to 0.5]) was reported by Vrijheid et al. 12 Divan et al., 13 in a study with more than 41 thosusand singletons, found no significant association between prenatal cell phone use and motor or cognitive/language developmental delays. The adjusted ORs were of 0.8 (95%CI: 0.7 to 1.0) and 1.1 (95%CI: 0.9 to 1.3) for cognitive/language in children 6 and 18 months old, respectively. The adjusted ORs were of 0.9 (95%CI: 0.8 to 1.1) and of 0.9 (95%CI: 0.8 to 1.0) for motor development delay in children 6 and 18 months old, respectively. Guxens et al., 14 in a cohort study with 2,618 children, reported a non-signiﬁcant association between behavioral problems and the number of calls (OR = 2.12; 95%CI: 0.95 to 4.74 for < 1 call/day; OR = 1.58; 95%CI: 0.69 to 3.60 for 1 to 4 calls/day; and OR = 2.04; 95%CI: 0.86 to 4.80 for ≥ 5 calls/day).

### The Effect of Maternal Cell Phone Use on Migraines and Headaches in Children

In a study conducted by Sudan et al., 15 the OR was of 30% (95%CI: 1.01 to 1.68) for migraines, and of 32% (95%CI: 1.23 to 1.40) for headache-related symptoms. It was higher for children with prenatal or postnatal exposure than for those with no exposure. Moreover, the OR was of 1.32 (95%CI:1.07 to 1.63), 1.77 (95%CI: 1.23 to 2.55), and 1.88 (95%CI: 1.21 to 2.77) for migraines (never used hands-free device, rarely used hands-free device, and often used hands-free device in children according to mother's report of cell phone use). 15

### Congenital Malformation

According to Baste et al., 16 the risk of congenital malformation was lower in children with medium (risk ratio [RR] = 0.99; 95%CI: 0.92 to 1.06) and high cell phone exposure (RR = 1.01; 95%CI: 0.92 to 1.11).

### Perinatal Mortality

In the study by Baste et al., 16 the risk of perinatal mortality was close to null in subjects with medium (RR = 0.89; 95%CI: 0.73 to 1.08) and high cell phone exposure (RR = 0.80; 95%CI: 0.60 to 1.06).

### Low Birth Weight

Still in the study by Baste et al., 16 the risk of low birth weight was close to null in subjects with medium (RR = 1.01; 95%CI: 0.92 to 1.10) and high cell phone exposure (RR = 1.02; 95%CI: 0.91 to 1.15). Lu et al. 3 compared birth weight between mothers who excessively or ordinarily utilized cell phones and found that the newborns of mothers who used cell phones excessivelly had a significantly lower birth weight ( *p* = 0.03). However, no significant difference was observed between the two groups regarding the proportion of low-birth weight newborns ( *p* = 0.6). 3

### Preterm Birth

Still in the study by Baste et al., 16 the risk of preterm birth was near null in subjects with medium (RR = 0.99; 95%CI: 0.92 to 1.06) and high cell phone exposure (RR = 1.01; 95%CI: 0.93 to 1.11). 16 However, no statistically significant differences were observed the groups of excessive and ordinary users of cell phones regarding the ratio of preterm birth ( *p* = 0.06).

### Small for Gestational Age Newborns

Still in the study by Baste et al., 16 the risk of having small for gestational age (SGA) newborns was close to null in subjects with medium (RR = 1.02; 95%CI: 0.96 to 1.09) and high (RR =1.03; 95%CI: 0.95 to 1.11) cell phone exposure compared with those with low exposure. Lu et al. 3 compared the ordinary and excessive use of cell phone by mothers, and reported that, in the latter group, the rates of lower birth weight and chest circumference ( *p* = 0.05) were significantly higher than those of the former group. However, no statistically significant differences were observed between the two groups regarding the birth height ( *p* = 0.792) and birth head circumference ( *p* = 0.06). 3

### Preeclampsia

Still in the study by Baste et al., 16 the risk of preeclampsia was lower among subjects with medium (RR = 0.89; 95%CI: 0.82 to 0.96) and high (RR = 0.89; 95%CI: 0.80 to 0.98) cell phone exposure as opposed to those with low exposure. 16

## Discussion

Divan et al. 7 found a significant association between behavioral problems at the age of 7 and prenatal and postnatal cell phone exposure. The results of a subsequent study by the same authors 10 on a larger sample size, conducted in 2012 after the consideration of additional confounders, showed that the previous finding was not coincidental. However, the OR was still smaller and remained significant. At ages as early as 6 to 12 months, no significant association was observed between prenatal cell phone use and motor or cognitive/language developmental delays. 13

There are many biological mechanisms behind the impact of in utero RFR-exposure on the brain of an infant. Exposure to RFR leads to energy transfer, thus elevating the permeability of the blood-brain barrier to macromolecules. The immature blood-brain barrier of the fetus can be susceptible even lower RFR energy induced by the mother's mobile phone use or the act of holding of the cell phone near the body that can affect the fetal brain. The lead in the mother's blood passes through the blood-placental barrier and penetrates the cord blood. The increased permeability of the blood-placental barrier due to RFR energy can result in the transmission of a high dose of lead. High levels of lead, a neurotoxin, in the cord blood can be transmitted to the fetal brain and provoke neurodevelopment complications. The release of melatonin by the pituitary gland can be impaired by RFR exposure. Also, the release of melatonin by the pituitary gland can be impaired by RFR exposure. The fetal stem cells, such as future neuronal cells, may be influenced by RFR exposure as well (Bellieni and Pinto, 2012). 17 Interestingly, there is no study to date that can confirm any of these hypotheses. 7

Wired-in hands-free kits (HFKs) can considerably reduce RFR exposure to the head, 18 which is inconsistent with the results of Sudan et al., 13 that found that the use of a hands-free device during pregnancy was associated with the increased risk. The highest ORs for migraines were found in the groups that often used hands-free devices, followed by those who “rarely used hands-free devices” and “never used hands-free devices.” 15 However, these differences between studies may be due to the fact that several factors that have been related to the infant, mother, or environmental factors can affect fetal growth, birth weight and gestation length.

According to a study by Ferraro et al., 19 college students who text excessively had higher levels of depression and anxiety and poor sleep quality. According to Lu et al., 3 pregnant women who used mobile phones in excess often slept later than those who used cell phones ordinarily. They suggested that anxiety, depression, and sleep problems, as indirect factors, may contribute to low birth weight. 3 Therefore, future research should should have sufficiently large samples to conduct a path analysis.

The present study has some strengths. Some of the previous research had long follow-up periods and large samples, such as the study by Papadopoulou et al., 4 which monitored 45,389 mother-child pairs over a period of 5 years. Their research was the largest on the association between maternal cell phone use and neurodevelopmental outcomes in children. 4 There are many shortcomings in the present systematic review that need to be addressed. First, the sample size of some studies was relatively small. 3 In several studies, no significant association was observed. In the study by Lu et al., 3 excessive cell phone use during pregnancy was not associated with low birth weight. However, there were only 16 infants with low birth weight in the study. 3 Therefore, the study may not have sufficient power to appropriately assess the association between excessive mobile phone use and low infant birth weight. Excessive mobile phone may be a risk factor for infant emergency transport but this, conclusion was conducted to be based on only 10 case (7 cases in ordinary cell phone users group and 3 cases in excessive cell phone users group). 3 The small sample size increases the chance of a false-positive (type-I) error. Second, all information gathered was self-reported by the participants, which may understimate the reliability of the responses. 3 The third limitation was related to the possibility of a recall bias, that is, the mother could have underestimated or overestimated the amount of cell phone use during pregnancy. However, a previous study 14 has shown that retrospectively reported phone calls are usually slightly underestimated. Also, pregnancy has a strong effect on the memories of mothers, so they are eager to remember accurately their behaviors within these unique days. 8 The fourth limitation is that almost all studies included in this systematic review reported the number of phone calls as an estimation. It seems that other factors like the extent of RF-EMF exposure are also important. Adverse in maternal, infant and child also depends on factors such as the duration of calls, the use of hands-free equipment, the communication system, and the frequency band. 14 The use of cell phones was also associated with smoking status, so that a higher level of smoking in the subjects link with to more calls. 19 However, one of the studies 20 has also assessed confounding variables. The fifth limitation is that almost all studies measure child neurodevelopment through subjective assessments of parental reports, except for one study 7 that had used expert examiners. Moreover, one study 4 adjusted important potential confounders by including sociodemographic characteristics, maternal personality, and psychological factors. However, it is unlikely that studies that did not report unmeasured confounding factors (such as, genetic or lifestyle factors) have affected our findings. Besides, the sample of unexposed groups was relatively small in most of studies. 7 12 14 Future studies should consider a sufficiently large sample of unexposed groups, although the rate of cell phone use is rapidly increasing. 21 The distribution of variables such as center area (one of the centers including: Cheonan, Seoul, Ulsan), age, and income was different regarding the subjects in the study by Choi et al., 7 but other general characteristics were identical. Finally, missing data was considered moderate, for example 33% of children had missing information related to emotional and behavioral problems at the age of 11. 8

## Conclusion

Studies on behavioral problems have reported different postnatal results, such as null findings among infants and a positive association in children.
